# Targeting tumor-initiating cells: Eliminating anabolic cancer stem cells with inhibitors of protein synthesis or by mimicking caloric restriction

**DOI:** 10.18632/oncotarget.3278

**Published:** 2015-02-10

**Authors:** Rebecca Lamb, Hannah Harrison, Duncan L. Smith, Paul A. Townsend, Thomas Jackson, Bela Ozsvari, Ubaldo E. Martinez-Outschoorn, Richard G. Pestell, Anthony Howell, Michael P. Lisanti, Federica Sotgia

**Affiliations:** ^1^ The Manchester Centre for Cellular Metabolism (MCCM), Institute of Cancer Sciences, University of Manchester, UK; ^2^ The Breakthrough Breast Cancer Research Unit, Institute of Cancer Sciences, University of Manchester, UK; ^3^ The Cancer Research UK Manchester Institute, University of Manchester, UK; ^4^ The Kimmel Cancer Center, Philadelphia, PA, USA

**Keywords:** tumor initiating cells, protein synthesis, puromycin, rapamycin, methionine restriction

## Abstract

We have used an unbiased proteomic profiling strategy to identify new potential therapeutic targets in tumor-initiating cells (TICs), a.k.a., cancer stem cells (CSCs). Towards this end, the proteomes of mammospheres from two breast cancer cell lines were directly compared to attached monolayer cells. This allowed us to identify proteins that were highly over-expressed in CSCs and/or progenitor cells. We focused on ribosomal proteins and protein folding chaperones, since they were markedly over-expressed in mammospheres. Overall, we identified >80 molecules specifically associated with protein synthesis that were commonly upregulated in mammospheres. Most of these proteins were also transcriptionally upregulated in human breast cancer cells *in vivo*, providing evidence for their potential clinical relevance. As such, increased mRNA translation could provide a novel mechanism for enhancing the proliferative clonal expansion of TICs. The proteomic findings were functionally validated using known inhibitors of protein synthesis, via three independent approaches. For example, puromycin (which mimics the structure of tRNAs and competitively inhibits protein synthesis) preferentially targeted CSCs in both mammospheres and monolayer cultures, and was ~10-fold more potent for eradicating TICs, than “bulk” cancer cells. In addition, rapamycin, which inhibits mTOR and hence protein synthesis, was very effective at reducing mammosphere formation, at nanomolar concentrations. Finally, mammosphere formation was also markedly inhibited by methionine restriction, which mimics the positive effects of caloric restriction in cultured cells. Remarkably, mammosphere formation was >18-fold more sensitive to methionine restriction and replacement, as directly compared to monolayer cell proliferation. Methionine is absolutely required for protein synthesis, since every protein sequence starts with a methionine residue. Thus, the proliferation and survival of CSCs is very sensitive to the inhibition of protein synthesis, using multiple independent approaches. Our findings have important clinical implications, since they may also explain the positive therapeutic effects of PI3-kinase inhibitors and AKT inhibitors, as they ultimately converge on mTOR signaling and would block protein synthesis. We conclude that inhibition of mRNA translation by pharmacological or protein/methionine restriction may be effective strategies for eliminating TICs. Our data also indicate a novel mechanism by which caloric/protein restriction may reduce tumor growth, by targeting protein synthesis in anabolic tumor-initiating cancer cells.

## INTRODUCTION

Tumor-initiating cells (TICs) are known to be resistant to many conventional therapies, and have been implicated in disease recurrence and metastatic spread [1-3]. Residual TICs are linked to poor patient survival in multiple tumor types. As TICs are extremely rare and represent only a small fraction of the total cancer cell population, we still know very little about what allows them to survive and propagate, especially under the harsh conditions associated with chemo- and radio-therapy [1-7].

Remarkably, TICs are thought to pheno-copy many of the characteristics of normal epithelial stem cells, such as immortalization, asymmetric cell division and resistance to stressors, such as DNA damage [2, 3, 7]. Hence, the term cancer stem cells (CSCs) is now used virtually interchangeably with TICs [1-7]. Another hallmark of epithelial TICs is their ability to grow under anchorage-independent conditions, when cultured using low-attachment plates [8]. Under these anchorage-independent growth conditions, CSCs/TICs spontaneously form 3D spheroid structures or “tumor-spheres”, that retain stem-like or progenitor cell properties. Conversely, under cell suspension conditions, most non-TICs undergo a form of apoptotic cell death, known as “anoikis”. It has been shown that each tumor-sphere is derived directly from the proliferative clonal expansion of a single TIC, and not from the aggregation of bulk cancer cells [8]. Furthermore, the enriched TIC population is more resistant to radiotherapy, showing enhanced DNA damage repair and lower levels of reactive oxygen species (ROS) [9]. As such, the preparation of tumor-spheres represents an efficient and convenient method to selectively enrich for TICs. When these tumor-spheres are specifically generated from primary breast cancer cells or cell lines, they are known as “mammospheres”.

Several mechanisms have been proposed to explain the increased resistance of TICs to clinical treatments. Firstly, both radiotherapy and the majority of chemotherapeutic treatments target rapidly dividing cells and it has been proposed that the resistance of TICs was due to them having a quiescent slow-cycling phenotype [10]. However, it has since been demonstrated that CSCs (at least in breast cancer) do cycle and their resistance to stress is not simply a function of quiescence [11, 12]. Furthermore, CSCs have a greater capacity to efflux chemotoxins due to an increased expression of ABC transporters possibly explaining their additional resistance to chemotoxins but obviously these cannot account radio-resistance [13]. CSCs have been shown to be resistance to apoptotic stimuli compared to their non-stem cell counter parts and to have an increased capacity for DNA damage repair and this now seems to be the most likely mechanism of radio- and chemo-resistance [14, 15]. Several studies have now shown that typical DNA damaging chemotherapeutic agents can even cause an upregulation of stem cell transcription factors and a direct conversion of cancer non-stem cells (bulk cells) into TICs, potentially increasing the TIC burden in patients rather than reducing it [16-19]. Thus, specifically targeting TIC populations in approaches that circumvent their resistance to DNA damaging therapy is a promising strategy for future cancer treatment.

To begin to understand the phenotypic behavior of TICs at a molecular level, we prepared large numbers of mammospheres from two different ER(+) breast cancer lines (MCF7 & T47D). The mammospheres were subjected to unbiased proteomic profiling to decipher their molecular composition and metabolic characteristics compared to the cells grown in monolayer. Based on proteomics analysis, we observed that mammospheres significantly upregulate molecules associated with protein synthesis, including ribosome-related proteins and protein-folding chaperones, as well as specific molecules involved in mRNA translation initiation, polypeptide elongation, tRNA synthesis and amino acid uptake. We speculate that TICs are highly anabolic and increase their capacity for protein synthesis, to drive their clonal expansion via cell proliferation. Treatment with well-established inhibitors of protein synthesis (puromycin, rapamycin or methionine restriction) directly validated that mammosphere formation is strictly dependent on nacent protein synthesis. Thus, our results may also explain the anti-cancer health benefits of caloric restriction, intermittent fasting and the vegetarian diet, by systemically reducing protein synthesis in TICs.

Currently, there is a tremendous need to identify a selective “Achilles' Heel” to eliminate TICs. Our new results highlight that TICs are especially functionally dependent on augmented protein synthesis, for their successful survival and continued propagation. This study provides a strong rationale for therapeutically targeting protein synthesis in the CSC population.

## RESULTS

### Greater than 70 ribosomal protein components, as well as an isoform of S6 Kinase (RPS6KB1), are upregulated in mammospheres

First, we performed unbiased label-free proteomic analysis on MCF7 cells, a commonly used ER-positive breast cancer cell line. Table 1 shows a non-redundant list of the 72 ribosome-related proteins that were selectively upregulated in MCF7 mammospheres, as directly compared with MCF7 cells derived from monolayers. Only proteins with a fold increase of ~1.5 or greater were selected for this analysis. Note that the expression levels of 21 large ribosomal proteins and 15 small ribosomal proteins were increased, as compared with monolayer cultures. Similarly, two large mitochondrial-specific ribosomal proteins were increased. A specific-isoform of ribosomal S6 kinase was also elevated (RPS6KB1) nearly 15-fold. Finally, 34 proteins involved in mRNA translation initiation, polypeptide elongation, tRNA synthesis and amino acid uptake, were all selectively upregulated in MCF7 mammospheres.

**Table 1:** Ribosomal-related Proteins Up-regulated in MCF7 Mammospheres

Symbol	Gene Description	Fold-Upregulation	ANOVA
**Small Subunit (14)**			
RPS9	40S ribosomal protein S9	Infinity	0.025
RPS3A	40S ribosomal protein S3A	582.09	1.12E-08
RPS27A	40S ribosomal protein S27A	32.14	0.0002
RPS3	40S ribosomal protein S3	28.39	6.36E-07
RPS23	40S ribosomal protein S23	19.85	5.84E-09
RPS20	40S ribosomal protein S20	7.55	2.73E-06
RPS7	40S ribosomal protein S7	6.85	4.56E-05
RPS21	40S ribosomal protein S21	5.74	0.006
RPS6	40S ribosomal protein S6	3.88	0.009
RPS4X	40S ribosomal protein S4, X-linked	3.69	0.02
RPS7	40S ribosomal protein S7	3.31	3.70E-07
RPS29	40S ribosomal protein S29	2.38	0.001
RPS9	40S ribosomal protein S9	2.03	0.002
RPS7	40S ribosomal protein S7	2.01	0.01
**Large Subunit (21)**			
RPL23A	60S ribosomal protein L23A	265.34	1.17E-10
RPLP0	60S acidic ribosomal protein P0	144.75	1.11E-05
RPL14	60S ribosomal protein L14	119.42	4.50E-09
RPL32	60S ribosomal protein L32	55.04	3.22E-08
RPL19	60S ribosomal protein L19	42.89	2.97E-09
RPL36	60S ribosomal protein L36	38.80	0.0002
RPL10	60S ribosomal protein L10	32.94	2.22E-07
RPL3	60S ribosomal protein L3	32.87	6.64E-06
RPL18A	60S ribosomal protein L18A	20.49	4.24E-11
RPL37A	60S ribosomal protein L37A	17.12	8.28E-07
RPL8	60S ribosomal protein L8	10.68	8.89E-06
RPL7	60S ribosomal protein L7	8.47	5.75E-08
RPL17	60S ribosomal protein L17	5.19	1.09E-06
RPL36AL	60S ribosomal protein L36A-like	3.59	0.0001
RPL5	60S ribosomal protein L5	3.56	0.0005
RPL6	60S ribosomal protein L6	3.00	4.91E-06
RPL12	60S ribosomal protein L12	2.91	0.002
RPL27	60S ribosomal protein L27	2.73	0.02
RPL9	60S ribosomal protein L9	2.18	0.01
RPL27A	60S ribosomal protein L27A	1.91	0.025
**Ribosomal S6 kinase (1)**			
RPS6KB1	Ribosomal protein S6 kinase, 70kDa, polypeptide 1	14.97	4.92E-09
**Mitochondrial Ribosomal Proteins (2)**			
MRPL45	39S ribosomal protein L45, mitochondrial	46.42	4.32E-11
MRPL17	39S ribosomal protein L17, mitochondrial	4.80	5.31E-05
**Translation initiation factors (required for mRNA binding to ribosomes) (7)**			
EIF4A1	Eukaryotic translation initiation factor 4A-I	439.74	4.07E-07
EIF4G3	Eukaryotic translation initiation factor 4 gamma 3	317.87	7.60E-09
EIF4G1	Eukaryotic translation initiation factor 4 gamma 1	66.29	2.02E-07
EIF5A2	Eukaryotic translation initiation factor 5A-2	37.14	2.49E-07
EIF3H	Eukaryotic translation initiation factor 3 subunit H	8.45	1.99E-06
EIF6	Eukaryotic translation initiation factor 6	5.23	2.74E-06
EIF3B	Eukaryotic translation initiation factor 3 subunit B (EIF3S9)	1.91	0.003
**Elongation factors (promote delivery of aminoacyl tRNAs to the ribosome) (8)**			
EEF1D	Elongation factor 1 delta	Infinity	1.41E-10
GFM1	Mitochondrial elongation factor G	97.22	1.32E-05
EEF1G	Elongation factor 1 gamma	17.00	0.008
EEF2	Elongation factor 2	15.67	0.03
EEF1A1	Elongation factor 1 alpha	7.56	9.90E-08
TSFM	Elongation factor Ts, mitochondrial	3.75	0.002
EEF1A2	Elongation factor 1-alpha 2	2.87	0.0002
TUFM	Elongation factor Tu, mitochondrial	2.31	0.0008
**Enzymes for tRNA Synthesis (18)**			
VARS	Valine--tRNA ligase	Infinity	3.38E-11
TRMT112	tRNA methyltransferase 112	362.30	3.19E-10
WARS	Tryptophan--tRNA ligase, cytoplasmic	272.93	2.51E-09
RARS	Arginine--tRNA ligase, cytoplasmic	154.98	2.70E-10
YARS	Tyrosine--tRNA ligase, cytoplasmic	77.20	1.90E-12
EPRS	Bifunctional-tRNA ligase (Glutamate and Proline)	60.55	1.60E-09
FARSA	Phenylalanine--tRNA ligase alpha	53.34	2.11E-08
AIMP1	Aminoacyl tRNA synthase complex-interacting multifunctional protein 1	20.28	0.0007
HARS2	Histidine--tRNA ligase, mitochondrial	13.05	4.16E-07
MARS	methionine-tRNA synthetase	6.77	7.67E-08
LARS	Leucine--tRNA ligase, cytoplasmic	6.63	6.99E-05
TARS	Threonyl-tRNA synthetase	6.34	0.001
RTCB	tRNA-splicing ligase (C22orf28)	3.52	2.05E-09
SARS	Serine--tRNA ligase, cytoplasmic	2.79	6.95E-06
NSUN2	tRNA (cytosine(34)-C(5))-methyltransferase	2.37	0.026
DARS	Aspartate--tRNA ligase, cytoplasmic	2.10	0.0009
GARS	Glycine--tRNA ligase	1.81	0.0003
AARS	Alanine--tRNA ligase, cytoplasmic	1.47	0.002
**Amino Acid Transporters (1)**			
SLC7A5	Solute carrier family 7 (Cationic amino acid transporter, y+ system), member 5	2.85	5.77E-06

**Figure 1:**
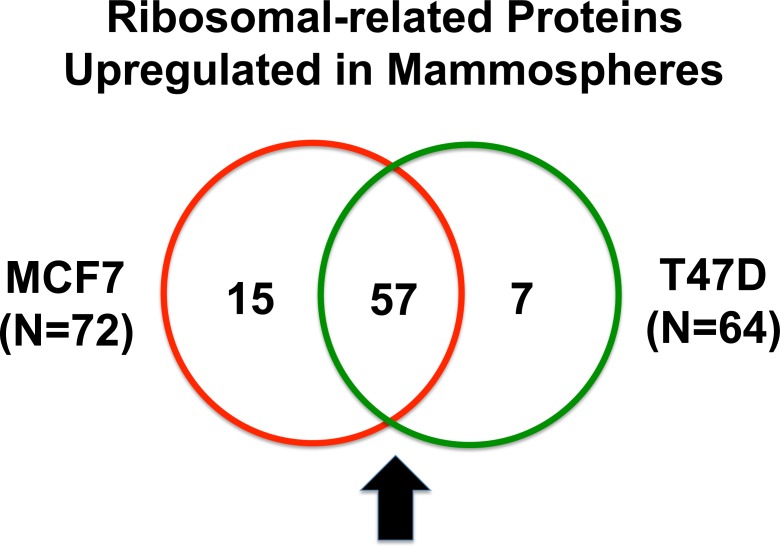
Venn diagram highlighting the conserved upregulation of ribosomal-related proteins in both MCF7 and T47D mammospheres Note that 57 ribosomal-related proteins were commonly upregulated in both data sets. These include proteins involved in ribosomal biogenesis, translation initiation, polypeptide elongation, tRNA synthesis and amino acid uptake.

For comparison purposes, we also performed unbiased label-free proteomic analysis on a second independent ER-positive breast cancer cell line, namely T47D cells. Our results are summarized in Table 2. Note that 64 ribosome-related proteins were specifically over-expressed in T47D mammospheres, as compared with T47D monolayer cultures processed in parallel. Remarkably, 57 of these proteins overlapped with the proteins that were upregulated in MCF7 mammospheres (57/64 = 89% overlap). See the Venn diagram presented in Figure 1.

**Table 2:** Ribosomal-related Proteins Up-regulated in T47D Mammospheres

Symbol	Gene Description	Fold-Upregulation	ANOVA
**Small Subunit (11)**			
**RPS9**	40S ribosomal protein S9	Infinity	0.03
**RPS3A**	40S ribosomal protein S3a	Infinity	5.42E-05
**RPS7**	40S ribosomal protein S7	8.22	0.006
**RPS21**	40S ribosomal protein S21	7.20	2.66E-07
RPS25	40S ribosomal protein S25	6.91	0.0002
**RPS23**	40S ribosomal protein S23	6.04	4.13E-11
**RPS20**	40S ribosomal protein S20	4.65	0.02
**RPS6**	40S ribosomal protein S6	3.49	4.54E-09
**RPS3**	40S ribosomal protein S3	3.01	0.048
**RPS4X**	40S ribosomal protein S4, X-linked	2.16	0.004
**Large Subunit (17)**			
**RPLP0**	60S acidic ribosomal protein P0	Infinity	5.13E-13
**RPL32**	60S ribosomal protein L32	30.62	4.74E-11
**RPL23A**	60S ribosomal protein L23A	21.67	5.20E-09
**RPL14**	60S ribosomal protein L14	13.89	1.55E-09
**RPL19**	60S ribosomal protein L19	9.42	2.13E-10
**RPL10**	60S ribosomal protein L10	6.72	0.03
**RPL18A**	60S ribosomal protein 18A	6.47	1.03E-11
**RPL36**	60S ribosomal protein L36	4.65	0.02
**RPL3**	60S ribosomal protein L3	3.96	0.00015
RPL23	60S ribosomal protein L23	3.43	0.004
**RPL8**	60S ribosomal protein L8	3.36	0.001
**RPL37A**	60S ribosomal protein L37A	2.76	6.45E-07
**RPL7**	60S ribosomal protein L7	2.65	0.004
**RPL5**	60S Ribosomal protein L5	2.21	0.001
**RPL3**	60S ribosomal protein L3	2.17	0.015
**RPL17**	60S ribosomal protein L17	2.04	0.007
RPL10L	60S ribosomal protein L10-like	1.92	0.01
**Ribosomal S6 kinase (1)**			
**RPS6KB1**	Ribosomal protein S6 kinase, 70kDa, polypeptide 1	4.82	5.72E-11
**Mitochondrial Ribosomal Proteins (4)**			
MRPL47	39S ribosomal protein L47, mitochondrial	10.69	0.03
**MRPL45**	39S ribosomal protein L45, mitochondrial	6.02	9.00E-11
**MRPL17**	39S ribosomal protein L17, mitochondrial	3.30	0.01
MRPS22	28S ribosomal protein S22, mitochondrial	2.12	0.0001
**Translation initiation factors (required for mRNA binding to ribosomes) (8)**			
**EIF4G3**	Eukaryotic translation initiation factor 4 gamma 3	107.50	0.001
**EIF5A2**	Eukaryotic translation initiation factor 5A-2	10.00	2.74E-07
**EIF4A1**	Eukaryotic translation initiation factor 4A-I	5.77	1.75E-13
**EIF3H**	Eukaryotic translation initiation factor 3 subunit H	3.33	1.26E-05
**EIF4G1**	Eukaryotic translation initiation factor 4 gamma 1	3.31	0.0001
EIF3E	Eukaryotic translation initiation factor 3 subunit E	2.02	5.87E-05
**EIF6**	Eukaryotic translation initiation factor 6	2.01	0.02
EIF2B4	Translation initiation factor eIF-2B subunit delta	2.00	0.001
**Elongation factors (promote delivery of aminoacyl tRNAs to the ribosome) (6)**			
**EEF1D**	Elongation factor 1 delta	30.44	3.10E-05
**EEF1G**	Elongation factor 1 gamma	4.93	0.003
**EEF1A1**	Elongation factor 1 alpha 1	4.40	3.21E-07
**EEF1A2**	Elongation factor 1 alpha 2	2.19	0.0008
**TUFM**	Elongation factor Tu, mitochondrial	2.03	6.98E-05
**EEF2**	Elongation factor 2	1.92	0.006
**Enzymes for tRNA Synthesis (16)**			
**LARS**	Leucine--tRNA ligase, cytoplasmic	Infinity	0.026
**TRMT112**	tRNA methyltransferase 112	39.44	0.0002
**VARS**	Valine--tRNA ligase	28.91	0.017
**AIMP1**	Aminoacyl tRNA synthase complex-interacting multifunctional protein 1	18.23	0.0001
**WARS**	Tryptophan--tRNA ligase, cytoplasmic	17.18	6.59E-11
**YARS**	Tyrosine--tRNA ligase, cytoplasmic	10.04	4.46E-10
**RARS**	Arginine--tRNA ligase, cytoplasmic	8.82	9.46E-10
**FARSA**	Phenylalanine--tRNA ligase alpha subunit	7.44	6.70E-08
**EPRS**	Bifunctional-tRNA ligase (Glutamate and Proline)	6.89	8.63E-09
**TARS**	Threonyl-tRNA synthetase	5.92	2.39E-08
**HARS2**	Histidine--tRNA ligase, mitochondrial	5.82	2.03E-07
**MARS**	Methionine-tRNA synthetase	3.24	2.32E-08
**RTCB**	tRNA-splicing ligase (C22orf28)	2.10	7.04E-05
**AARS**	Alanine--tRNA ligase, cytoplasmic	2.09	1.47E-05
**SARS**	Serine--tRNA ligase, cytoplasmic	1.82	0.001
**NSUN2**	tRNA (cytosine(34)-C(5))-methyltransferase	1.67	0.027
**Amino Acid Transporters (1)**			
**SLC7A5**	Solute carrier family 7 (Cationic amino acid transporter, y+ system), member 5	2.02	0.004

**Proteins shown in BOLD were also up-regulated in MCF7 Mammospheres. Others that were unique to T47D mammospheres are underlined.**

### Heat shock proteins (HSPs)/protein-folding chaperones are upregulated in MCF7 and T47D mammospheres

**Figure 2:**
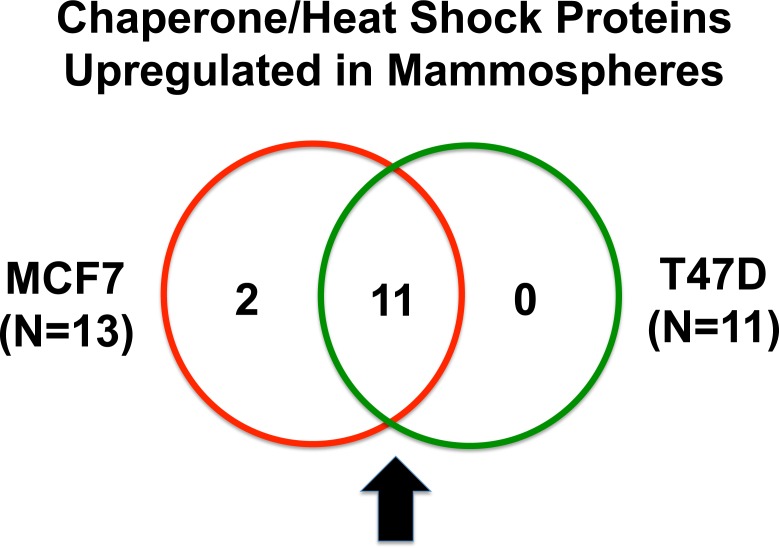
Venn diagram highlighting the conserved upregulation of heat shock proteins/molecular chaperones in both MCF7 and T47D mammospheres Note that 11 heat shock proteins, involved in protein folding, were commonly upregulated in both data sets.

Heat shock proteins are important for proper protein folding during protein synthesis. Table 3 shows a list of 13 heat shock proteins that were selectively upregulated in MCF7 mammospheres, relative to MCF7 monolayers. Note that 2 of these heat shock proteins are mitochondrial-specific chaperones (HSPA9 and HSPD1). Similarly, 11 heat shock proteins were specifically over-expressed in T47D mammospheres, as compared with T47D monolayer cultures processed in parallel (Table 4). Importantly, all eleven proteins overlapped with the proteins that were upregulated in MCF7 mammospheres (11/11 = 100% overlap). See the Venn diagram presented in Figure 2.

**Table 3:** Heat Shock Proteins Up-regulated in MCF7 Mammospheres

Symbol	Gene Description	Fold-Upregulation	ANOVA
HSPA1A	Heat shock 70 kDa protein 1A/1B	Infinity	2.24E-13
HSPA9	Stress-70 protein, mitochondrial	298,325.4	2.62E-13
HSPA4L	Heat shock 70 kDa protein 4L	172.50	2.84E-10
HSP90AA1	Heat shock protein HSP 90-alpha	116.77	3.20E-10
AHSA1	Activator of 90 kDa heat shock protein ATPase homolog 1	40.75	0.001
HSPH1	Heat shock protein 105 kDa	35.80	0.0002
HSPD1	60 kDa heat shock protein, mitochondrial	16.45	0.0001
HSPA1L	Heat shock 70 kDa protein 1-like	14.45	1.52E-10
HSPB1	Heat shock protein beta-1	7.57	7.30E-08
HSP90AB1	Heat shock protein HSP 90-beta	4.65	1.09E-06
HSP90B1	Heat shock protein GRP94	4.43	0.006
HSPA8	Heat shock cognate 71 kDa protein	3.59	3.34E-06
HSPA4	Heat shock 70 kDa protein 4	2.44	0.005

**Table 4:** Heat Shock Proteins Up-regulated in T47D Mammospheres

Symbol	Gene Description	Fold-Upregulation	ANOVA
**HSPD1**	60 kDa heat shock protein, mitochondrial	69.06	1.45E-05
**HSPA1A**	Heat shock 70 kDa protein 1A/1B	43.00	4.58E-05
**AHSA1**	Activator of 90 kDa heat shock protein ATPase homolog 1	20.41	0.001
**HSP90AA1**	Heat shock protein HSP 90-alpha	17.79	3.81E-11
**HSPB1**	Heat shock protein beta-1	14.63	1.74E-05
**HSPA4**	Heat shock 70 kDa protein 4	9.53	2.48E-07
**HSPA4L**	Heat shock 70 kDa protein 4L	9.26	2.40E-09
**HSPH1**	Heat shock protein 105 kDa	5.17	0.0004
**HSPA9**	Stress-70 protein, mitochondrial	4.14	0.03
**HSPA1L**	Heat shock 70 kDa protein 1-like	3.35	1.19E-09
**HSP90AB1**	Heat shock protein HSP 90-beta	2.11	0.01

**Proteins shown in BOLD were also up-regulated in MCF7 Mammospheres.**

Taken together, our results predict that protein synthesis may be critical for the survival and propagation of cancer stem cells and/or progenitor cells.

### Functional effects of puromycin, a known inhibitor of protein synthesis, on mammosphere formation

**Figure 3:**
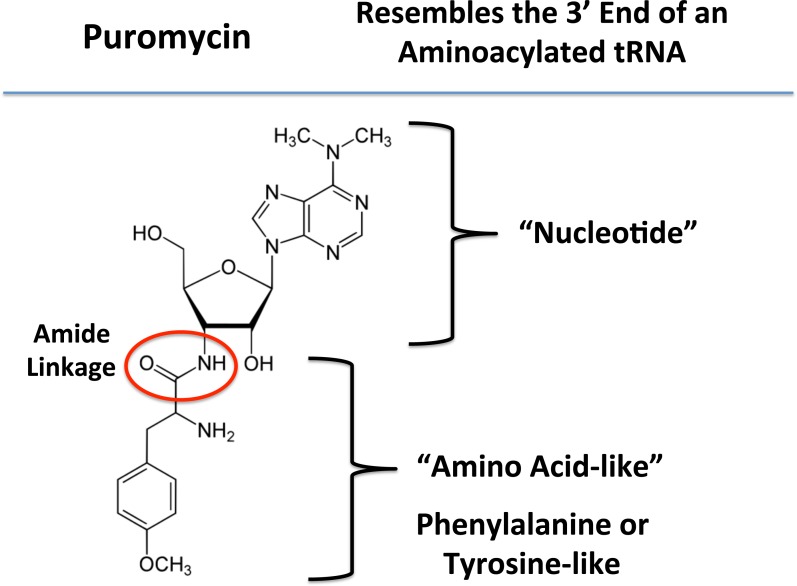
Puromycin: Structure and key features Puromycin resembles the 3′ end of an aminoacylated tRNA, which interacts with the A-site of the ribosome. During protein synthesis, puromycin transfers to the growing polypeptide chain, leading to the generation of a puromycylated-peptide, which is prematurely released. As such, puromycin inhibits protein synthesis via a premature chain termination mechanism.

Next, to functionally validate the hypothesis that mammosphere formation strictly requires protein synthesis, we used a highly-specific inhibitor that mimics the nucleotide-polypeptide linkage that occurs in tRNAs, namely puromycin [20]. By mimicking the structure of tRNAs, puromycin competitively inhibits protein synthesis. Also, puromycin is physically transferred to the growing polypeptide chain, leading to the generation of puromycylated-peptides, which are prematurely released (Figure 3). Thus, puromycin inhibits protein synthesis via a premature chain termination mechanism [20].

**Figure 4:**
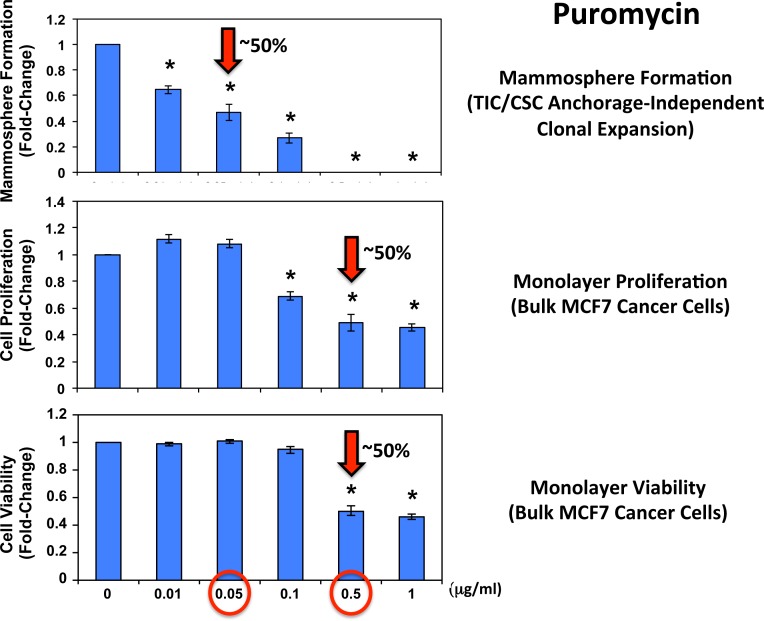
Puromycin significantly reduces mammosphere formation in MCF7 cells, without affecting MCF7 cell viability or proliferation Increasing concentrations of puromycin inhibit mammosphere formation, using an ER-positive cell line (MCF7). Importantly, puromycin significantly reduces mammosphere formation, with an IC-50 of ~ 0.05 μg/ml. However, mammosphere formation was completed abolished at 0.5 μg/ml. The vehicle-alone control was normalized to one. (*)p <0.05.

Figure 4 shows the effects of increasing concentrations of puromycin on mammosphere formation, using an ER-positive breast cancer cell line (MCF7). Importantly, puromycin significantly reduced mammosphere formation, with an IC-50 of ~ 0.05 μg/ml and mammosphere formation was completed abolished at 0.5 μg/ml.

In striking contrast, monolayer MCF7 cells were ~10 times less sensitive to the effects of puromycin. Puromycin reduced both the i) proliferation and ii) viability of monolayer MCF7 cells, with an IC-50 of ~ 0.5 μg/ml, a concentration 10-times higher than the IC-50 for mammosphere formation (Figure 4). In addition, mammosphere formation was inhibited by >70% at a concentration of 0.1 μg/ml; this same concentration had no effect on the viability of monolayer MCF7 cells.

**Figure 5:**
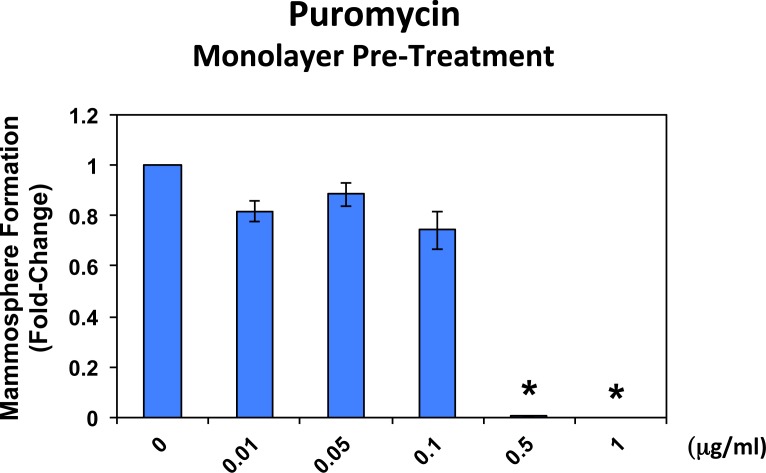
Puromycin pre-treatment of MCF7 cell monolayers completely prevents mammosphere formation When monolayer MCF7 cells were pre-treated with puromycin for 4 days and then trypsinized and plated for mammosphere assays (in the absence of puromycin), mammosphere forming activity was completely abolished by puromycin pre-treatment at 0.5 μg/ml. At this same concentration, nearly 50% of the monolayer cells still remains viable and proliferate (See Figure 4).

In addition, when monolayer MCF7 cells were pre-treated with puromycin for 4 days and then trypsinized and plated for mammosphere assays (in the absence of puromycin), mammosphere forming activity was completely eliminated by puromycin pre-treatment at 0.5 μg/ml (Figure 5). At this same concentration, ~50% of monolayer MCF7 cells still remain viable and proliferate (Figure 4). This indicates that protein synthesis inhibitors can preferentially target TICs, even in the presence of adjacent non-TIC cells, in the setting of an attached monolayer.

Thus, we conclude that TICs/CSCs are clearly more sensitive to the functional effects of inhibiting protein synthesis, directly supporting the results of our unbiased proteomics analysis.

### Functional effects of rapamycin and methionine restriction on mammosphere formation

**Figure 6:**
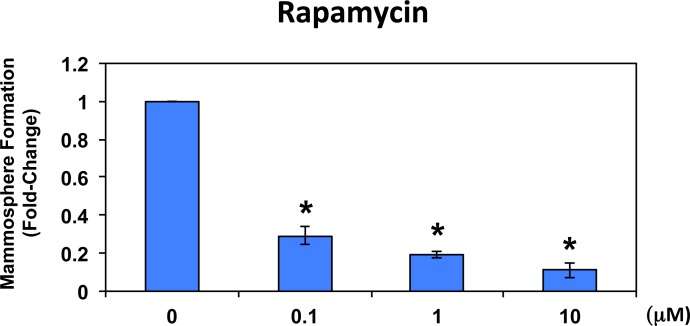
Rapamycin significantly reduces mammosphere formation in MCF7 cells Note that rapamycin also effectively reduces mammosphere formation in this cellular context, with an IC-50 of <100 nM. The vehicle-alone control was normalized to one. (*)p <0.05.

To further validate that mammosphere formation is functionally dependent on protein synthesis, we next used a well-established FDA-approved drug that potently inhibits protein synthesis, namely rapamycin [21-23]. Figure 6 shows the effects of increasing concentrations of rapamycin on mammosphere formation. Note that rapamycin significantly reduces mammosphere formation in MCF7 cells, with an IC-50 < 100 nM.

**Figure 7:**
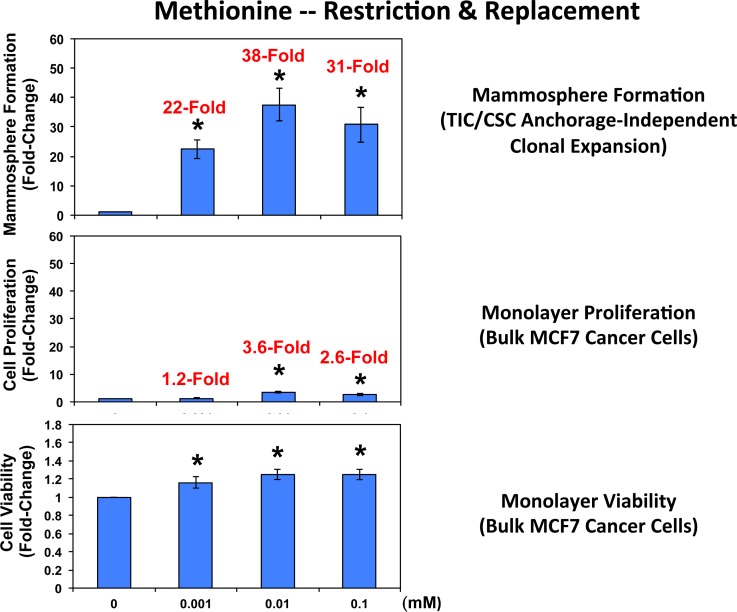
Methionine restriction significantly reduces mammosphere formation in MCF7 cells Note that mammosphere formation was dramatically inhibited by methionine deprivation. Importantly, mammosphere formation was functionally restored to normal levels by the dose-dependent re-addition of methionine to the methionine-free culture media, with maximal effects occurring at 0.01 mM (10 μM). Mammosphere media normally contains 0.1 mM (100 μM). Parallel experiments with MCF7 cells grown as monolayer cultures are shown for comparison. Interestingly, mammosphere growth is 10-to18-fold more sensitive to the effects of methionine-restriction and replacement. (*)p <0.05.

Complementary results were obtained with methionine restriction. Methionine is absolutely required for protein synthesis, since every new protein sequence starts with a methionine residue [24-26]. Note that mammosphere formation was dramatically inhibited by methionine deprivation (Figure 7), which mimics the positive effects of caloric restriction in cultured cells. Importantly, mammosphere formation was functionally restored to normal levels by the dose-dependent re-addition of methionine to the methionine-free culture media, with maximal effects occurring at 0.01 mM (10 μM). It should be noted that mammosphere media normally contains 0.1 mM methionine (100 μM).

Remarkably, mammosphere formation appeared to be >18-times more sensitive to the effects of methionine deprivation and replacement, when compared directly with MCF7 cell monolayers (Figure 7). More specifically, the re-addition of 0.001 mM methionine stimulated MCF7 mammosphere formation by 22-fold; in contrast, the same concentration of methionine only stimulated MCF7 monolayer proliferation by 1.2-fold (Figure 7). Similar results were also obtained at higher concentrations of methionine (0.01 and 0.1 mM), with mammospheres again showing a >10-fold increase relative to monolayers.

**Figure 8:**
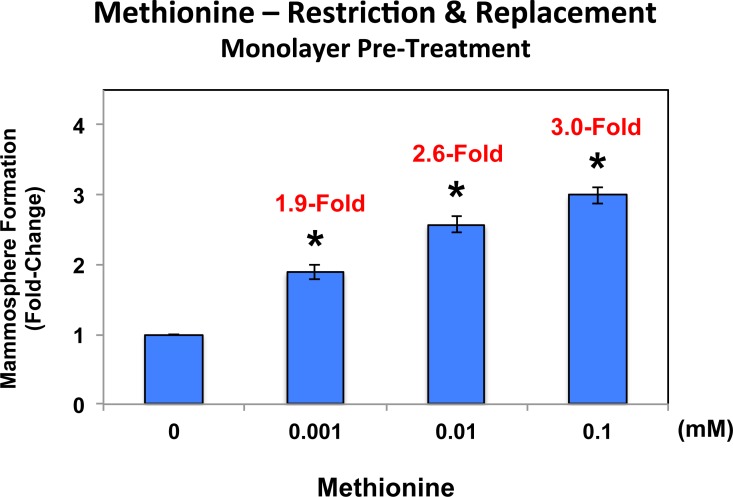
Pre-treatment of MCF7 cell monolayers with methionine restriction significantly reduces mammosphere formation MCF7 cell monolayers were pre-treated with various concentrations of methionine (0, 0.001, 0.01 and 0.1 mM) for 4 days and then trypsinized and re-plated for mammosphere assays (in the presence of normal levels of methionine (0.1 mM)). Note that under these conditions, mammosphere-forming activity was significantly reduced by up to 3-fold (compare 0 vs. 0.1 mM). As such, methionine restriction is also effective at reducing “stemness” in the context of MCF7 cell monolayers.

In addition, MCF7 cell monolayers were pre-treated with methionine at different concentrations (0, 0.001, 0.01 and 0.1 mM) for 4 days and then trypsinized and re-plated for mammosphere assays (in the presence of normal levels of methionine (0.1 mM)). Under these conditions, mammosphere-forming activity was significantly reduced by up to 3-fold (Figure 8; compare 0 vs. 0.1 mM). Importantly, methionine restriction did not affect MCF7 monolayer viability (as seen in Figure 7). Thus, methionine restriction is also effective at reducing “stemness” in the context of MCF7 cell monolayers.

Therefore, pharmacological inhibition of protein synthesis and methionine depletion (as a mimetic of caloric restriction) may be effective strategies for eliminating cancer stem cells [22-28].

### Relevance of protein synthesis related targets in human breast cancers

To assess the clinical relevance of our results, we also determined whether the proteomic targets that we identified in mammospheres were transcriptionally over-expressed in human breast cancer cells *in vivo*. Towards this end, we exploited a clinical data set of tumor samples from 28 breast cancer patients [29, 30]. These tumor samples were subjected to laser-capture micro-dissection, to separate epithelial cancer cells from adjacent stroma. Tables 5 and 6 present a summary of these findings. Overall, 60 proteomic targets that we identified in mammospheres were also transcriptionally elevated in human breast cancer cells *in vivo* and the majority of these targets were also upregulated either in MCF7 and/or T47D mammospheres. As such, the new protein targets that we identified in mammospheres may be especially relevant for improving human breast cancer diagnosis and therapy.

**Table 5:** “Ribosomal Targets” Over-Expressed in Mammospheres are also Transcriptionally Up-regulated in Human Breast Cancer Cells In Vivo (Cancer Epithelia vs. Tumor Stroma)

Symbol	Gene Description	Fold-Upregulation (Epithelial/Stromal)	P-value
**Small Subunit (12)**			
RPS27A	40S ribosomal protein S27A	4.63	1.19E-05
RPS3A	40S ribosomal protein S3a	4.59	1.35E-05
RPS20	40S ribosomal protein S20	4.44	2.25E-05
RPS6	40S ribosomal protein S6	4.09	7.27E-05
RPS25	40S ribosomal protein S25	4.05	8.29E-05
RPS4X	40S ribosomal protein S4, X-linked	3.92	1.27E-04
RPS23	40S ribosomal protein S23	3.56	3.92E-04
RPS7	40S ribosomal protein S7	3.55	4.06E-04
RPS3	40S ribosomal protein S3	3.33	7.91E-04
RPS21	40S ribosomal protein S21	2.75	4.00E-03
RPS29	40S ribosomal protein S29	2.31	1.25E-02
RPS9	40S ribosomal protein S9	1.83	3.65E-02
**Large Subunit (20)**			
RPL7	60S ribosomal protein L7	5.21	1.53E-06
RPL23A	60S ribosomal protein L23A	5.13	1.99E-06
RPL3	60S ribosomal protein L3	5.01	3.14E-06
RPL10	60S ribosomal protein L10	4.91	4.48E-06
RPLP0	60S acidic ribosomal protein P0	4.89	4.71E-06
RPL14	60S ribosomal protein L14	4.45	2.15E-05
RPL17	60S ribosomal protein L17	4.15	6.00E-05
RPL32	60S ribosomal protein L32	4.03	8.74E-05
RPL6	60S ribosomal protein L6	4.00	9.86E-05
RPL19	60S ribosomal protein L19	3.97	1.05E-04
RPL8	60S ribosomal protein L8	3.86	1.51E-04
RPL9	60S ribosomal protein L9	3.71	2.49E-04
RPL23	60S ribosomal protein L23	3.56	3.92E-04
RPL12	60S ribosomal protein L12	3.42	6.09E-04
RPL36	60S ribosomal protein L36	3.37	6.91E-04
RPL37A	60S ribosomal protein L37A	3.13	1.42E-03
RPL27	60S ribosomal protein L27	2.57	6.41E-03
RPL18A	60S ribosomal protein L18A	2.28	1.34E-02
RPL5	60S ribosomal protein L5	1.86	3.45E-02
RPL36AL	60S ribosomal protein L36A-like	1.70	4.76E-02
**Mitochondrial Ribosomal Proteins (2)**			
MRPS22	28S ribosomal protein S22, mitochondrial	3.27	9.31E-04
MRPL17	39S ribosomal protein L17, mitochondrial	2.94	2.38E-03
**Translation initiation factors (required for mRNA binding to ribosomes) (4)**			
EIF3H	Eukaryotic translation initiation factor 3 subunit H	4.70	9.25E-06
EIF3E	Eukaryotic translation initiation factor 3 subunit E	3.57	3.75E-04
EIF2B4	Translation initiation factor eIF-2B subunit delta	3.01	1.97E-03
EIF3B	Eukaryotic translation initiation factor 3 subunit B (EIF3S9)	2.20	1.59E-02
**Elongation factors (promote delivery of aminoacyl tRNAs to the ribosome) (5)**			
EEF2	Elongation factor 2	4.01	9.29E-05
EEF1G	Elongation factor 1 gamma	3.71	2.44E-04
TUFM	Elongation factor Tu, mitochondrial	3.38	6.74E-04
EEF1A1	Elongation factor 1 alpha	3.16	1.30E-03
EEF1D	Elongation factor 1 delta	2.50	7.67E-03
**Enzymes for tRNA Synthesis (7)**			
RTCB	tRNA-splicing ligase (C22orf28)	4.58	1.37E-05
MARS	methionine-tRNA synthetase	4.35	3.00E-05
EPRS	Bifunctional-tRNA ligase (Glutamate and Proline)	4.06	8.10E-05
DARS	Aspartate--tRNA ligase, cytoplasmic	3.43	5.87E-04
WARS	Tryptophan--tRNA ligase, cytoplasmic	2.48	8.17E-03
SARS	Serine--tRNA ligase, cytoplasmic	2.15	1.81E-02
YARS	Tyrosine--tRNA ligase, cytoplasmic	1.72	4.55E-02

**-Transcriptional profiling data derived from the analysis of N=28 breast cancer patients are shown, high-lighting the levels of fold-upregulation observed in the epithelial cancer cell compartment (relative to the tumor stroma), and corresponding p-values derived from the analysis of these clinical samples.**

**-Proteins listed above (50 in total) were all upregulated either in MCF7 or T47D mammospheres (See Tables 1 & 2).**

**Table 6:** Heat Shock Proteins Over-Expressed in Mammospheres are also Transcriptionally Up-regulated in Human Breast Cancer Cells In Vivo (Cancer Epithelia vs. Tumor Stroma)

Symbol	Gene Description	Fold-Upregulation (Epithelial/Stromal)	P-value
**(Epithelial/Stromal)**			
HSP90AB1	Heat shock protein HSP 90-beta	4.94	4.03E-06
HSP90AA1	Heat shock protein HSP 90-alpha	3.76	2.12E-04
HSPA4	Heat shock 70 kDa protein 4	3.75	2.18E-04
HSPA9	Stress-70 protein, mitochondrial	3.69	2.64E-04
HSPB1	Heat shock protein beta-1	3.27	9.51E-04
HSPD1	60 kDa heat shock protein, mitochondrial	3.42	5.93E-04
HSPH1	Heat shock protein 105 kDa	3.18	1.22E-03
HSPA8	Heat shock cognate 71 kDa protein	3.11	1.49E-03
AHSA1	Activator of 90 kDa heat shock protein ATPase homolog 1	2.49	7.88E-03
HSP90B1	Heat shock protein 90kDa beta (Grp94), member 1	2.43	9.33E-03

**-Transcriptional profiling data derived from the analysis of N=28 breast cancer patients are shown, high-lighting the levels of fold-upregulation observed in the epithelial cancer cell compartment (relative to the tumor stroma), and corresponding p-values derived from the analysis of these clinical samples.**

**-Proteins listed above (10 in total) were all upregulated either in MCF7 or T47D mammospheres (See Tables 3 & 4).**

## DISCUSSION

**Figure 9:**
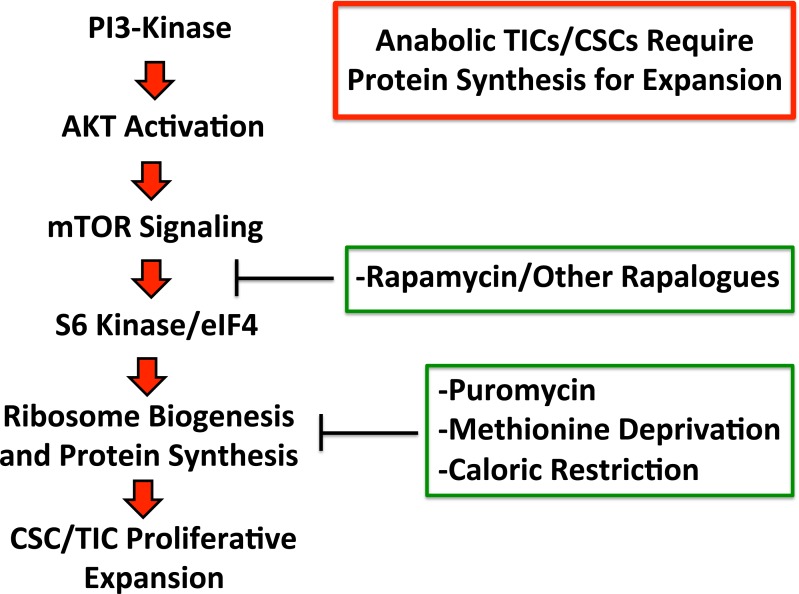
Anabolic tumor-initiating cells require protein synthesis for clonal expansion Activation of the PI3-kinase/AKT/mTOR signaling pathway is known to converge on ribosomal biogenesis and protein synthesis. Thus, the therapeutic effects of PI3-kinase inhibitors and AKT inhibitors, may be explained by their ability to inhibit mTOR signaling and block protein synthesis in TICs. Similarly, direct pharmacological inhibition of protein synthesis and/or caloric restriction or protein restriction may have similar beneficial therapeutic effects. Thus, our current data provide a novel convergent mechanism by which inhibitors of PI3-kinase, AKT, mTOR, as well as caloric restriction, may all directly target tumor-initiating cells, by inhibiting protein synthesis.

Here, using unbiased label-free proteomics analysis, we show that the cells of mammospheres (a population which is enriched for TICs and other progenitor cells) functionally overexpress numerous proteins, related to protein synthesis, including ribosomal biogenesis, mRNA translation initiation, polypeptide elongation, tRNA synthesis, amino acid uptake and protein folding. The potential clinical relevance of these targets was further validated using a previously published data set of human breast cancer samples (N=28 patients), that were subjected to laser-capture microdissection, to separate the epithelial tumor cells from the adjacent tumor stroma. Thus, these novel anabolic targets reveal a metabolic “Achilles' Heel” to allow the elimination of CSCs. In accordance with this idea, we demonstrate that the therapeutic targeting of protein synthesis in mammospheres (via puromycin, rapamycin or methionine-restriction) is indeed sufficient to prevent their proliferative expansion, as assessed using mammosphere formation as a functional assay (summarized schematically in Figure 9). In accordance with our results, a recent paper has shown that the mTOR inhibitor Torin-1 selectively targets human colon CSCs [31].

**Figure 10:**
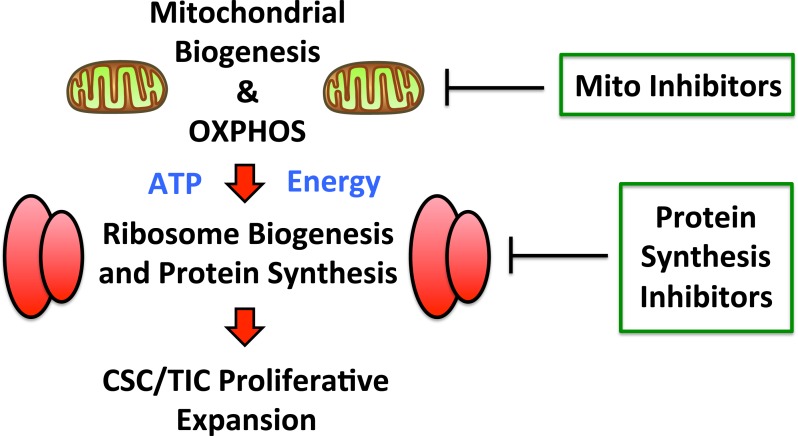
Augmented mitochondrial OXPHOS may help fuel increased protein synthesis Recently, we showed that mitochondrial oxidative metabolism is significantly amplified in mammospheres, as evidenced by i) unbiased proteomics analysis and ii) functional validation with inhibitors of mitochondrial OXPHOS. As such, enhanced mitochondrial energy production could help directly “fuel” increased protein synthesis in CSCs, thereby driving and maintaining the anabolic phenotype of TICs. Thus, inhibition of mitochondrial function and protein synthesis may both be beneficial.

Recently, we also reported that mitochondrial oxidative metabolism is also markedly amplified in mammospheres, as evidenced by i) unbiased proteomics analysis and ii) functional validation with inhibitors of mitochondrial OXPHOS [32]. Thus, enhanced mitochondrial energy production could help directly “fuel” increased protein synthesis in CSCs, thereby driving and maintaining the anabolic phenotype of TICs (Figure 10).

### Deregulation of protein synthesis

Deregulation of protein synthesis is a relatively unexplored but emerging mechanism of cancer progression. Two of the better documented examples of this phenomenon are increased protein synthesis as a result of c-MYC and mTOR oncogenic signaling. Although better known for targeting genes involved in cell cycle regulation, it has been known for some time that c-MYC directly targets multiple components of the translational machinery including: RNA polymerases I, II and III; ribosomal proteins; translation initiation factors; elongation factors; and rRNA [33-38]. Until more recently, the consequence and importance of this protein synthesis up regulation has remained unknown and the complexity and breadth of c-MYC targets has made it a difficult question to address [39]. Specific ribosomal protein haploinsufficiency (L24^+/−^ and L38^+/−^) have recently helped to address the role of c-MYC induced protein synthesis [40]. It was thus demonstrated ribosomal protein haploinsufficiency is able to rescue mice from increased rates of protein synthesis downstream of oncogenic Eμ-Myc signaling. In these mice, the growth of Myc-overexpressing B cells was returned to normal and remarkably this was coupled with the restoration of cell division rates to near wild-type levels [40]. These results imply that c-MYC directly couples cell growth and cell division, at least in part, by a deregulation of protein synthesis. Furthermore, the oncogenic potential of c-MYC was strongly impaired by ribosomal protein haploinsufficiency genetic backgrounds, with the onset of lymphomas being dramatically delayed Eμ-Myc;L24^+/−^ and Eμ-Myc;L38^+/−^ mice compared to Eμ-Myc [40]. This may be due to an increase in the apoptotic response to Myc oncogenic activity observed with ribosomal protein haplo-insufficiency genetic backgrounds.

Unlike c-MYC, the oncogenic mTOR signaling pathway is readily associated with the control of protein synthesis, targeting mRNA translation and ribosome biogenesis [41-48]. The signaling cascade initiates with PI3-kinase producing phosphatidylinositol-3,4-bisphosphate and phosphatidylinositol-3,4,5-triphosphate which act as second messages or as docking sites for the serine/threonine kinase Akt [49]. Recruitment of Akt to PI3-kinase products allows Akt-phosphorylation and thus activated, by the kinase PDK1. Activated Akt subsequently targets mTOR, in turn promoting translation via the activation of p70 S6 kinase (S6K) and the initiation factor 4E [50, 51]. S6K phosphorylates ribosomal protein S6 and promotes the translation of translational machinery [52].

### Methionine and calorie restriction

Methionine-free medium reduced the proliferation and viability of the cells in mammospheres. Reduction of methionine also reduces proliferation and increases apoptosis of embryonal stem cells (ESC) whilst apparently not affecting more differentiated cells [53]. ESC can be induced to form differentiated cardiac cells and a methionine free medium can be used to remove remaining ESCs which, if transplanted with cardiac cells form teratomas [54]. Our data is the first suggestion that epithelial TICs may be similarly sensitive to methionine depletion. It is well known that tumor cell lines and primary tumors in-vitro and when grown in-vivo in rodents are growth inhibited by methionine depletion whereas normal cells are unresponsive. Methionine restriction (MR) was shown to reduce the growth of sarcomas [55, 56] [57], adenocarcinomas [25, 58] and mammary tumors in rodents [59], as well as of human tumor cell lines [60], human primary tumors in-vitro [61] and tumor growth in nude mice [62, 63].

Low methionine diets in rodents produce similar effects as calorie restriction [64]. Tumor formation and tumor growth is reduced and lifespan is increased although MR does not appear as potent as calorie restriction or general protein restriction [26]. MR may produce longevity effects, in part, acting through reduction of the formation of free radicals in mitochondria [64, 65] and, in part, by inhibition of protein synthesis in precursor cells as demonstrated in this report. Also, methionine restriction increases the stress tolerance of human fibroblasts, reduces senescence and increases their doubling time [66, 67]. In mammals, the effect of MR appears to be growth hormone dependent [68].

Vegans and vegetarians have relatively low intakes of methionine compared with meat eaters and some but not all recent studies suggest that vegans have lower cancer and cardiovascular disease risk [69-71]. It has been suggested that low-methionine vegan diets may be used as a feasible approach for life extension [72]. Ornish and his colleagues [73] were able to show that the concentration of prostate specific antigen (PSA) was reduced in men with prostate cancer treated only with lifestyle changes, which included a vegan diet and exercise. A recent phase I study demonstrated that methionine restriction was well tolerated for up to 17 weeks in patients with various solid tumors [74].

## CONCLUSIONS

In conclusion, based on our current analysis using mammosphere cultures, we propose that inhibition of protein synthesis is a new therapeutic strategy for eradicating TICs, to potentially prevent tumor recurrence, metastasis and poor clinical outcome in breast cancer patients. This strategy might also be extended to other tumor types, as many of the phenotypic features of TICs are highly conserved between different epithelial cancer types.

## MATERIALS AND METHODS

### Materials

Breast cancer cell lines (MCF7 and T47D) were purchased from the ATCC. Puromycin, rapamycin, methionine and methionine-free media were obtained commercially from Sigma-Aldrich. Gibco-brand cell culture media (DMEM/F12) was purchased from Life Technologies.

### Monolayer culture

50,000 cells were plated in normal medium (DMEM, 10% FCS, L-glutamine, supplemented with Pen-Strep) for 24hr, followed by treatment with increasing concentrations of a specific inhibitor (puromycin) or nutrient (methionine) for a further 4 days. Cells were then collected by trypsinization and centrifugation. To quantitatively determine cell growth, the number of cells after dtreatment was counted using an automatic cell counter (Biorad) and differences compared to untreated cells was calculated and expressed as fold-change. To assess cell viability, cells were incubated for 1 minute with Trypan Blue (Sigma, #T8145) using a 1:1 ratio. The number of Trypan Blue positive cells (non-viable) was measured using an automatic cell counter (Biorad) and compared to untreated controls. For puromycin treatments, cells were also plated into mammosphere culture to assess stem cell-like activity with no further drug treatment. All monolayer experiments were performed in triplicate, three times independently, such that each data point represents the average of 9 replicates.

### Mammosphere culture

To directly assess the effects of specific inhibitors (puromycin/rapamycin) or nutrients (methionine) on mammosphere formation, cultures were supplemented with increasing concentrations of puromycin, rapamycin or methionine, as indicated in a given experiment. A single cell suspension was prepared using enzymatic (1x Trypsin-EDTA, Sigma Aldrich, #T3924), and manual disaggregation (25 gauge needle) to create a single cell suspension [8, 32]. Cells were plated at a density of 500 cells/cm^2^ in mammosphere medium (DMEM-F12/B27/20ng/ml EGF/PenStrep) in non-adherent conditions, in culture dishes coated with (2-hydroxyethylmethacrylate) (poly-HEMA, Sigma, #P3932). Cells were grown for 4-to-5 days and maintained in a humidified incubator at 37°C at an atmospheric pressure in 5% (v/v) carbon dioxide/air. After 5 days for culture, spheres >50 μm were counted using an eye piece graticule, and the percentage of cells plated which formed spheres was calculated and is referred to as percentage mammosphere formation, and was normalized to one (1 = 100 %MSF). All mammosphere experiments were performed in triplicate, three times independently, such that each data point represents the average of 9 replicates.

### Methionine restriction and replacement

For methionine restriction experiments, DMEM-F12 was replaced with methionine-free DMEM (Gibco, #21013-24) and supplemented with 30mg/L cysteine (Sigma, #C7352-25G).

### Label-free quantitative proteomics analysis

For proteomic analysis, mammospheres were collected by centrifugation at 800 rpm for 10 minutes. Cell lysates were prepared for trypsin digestion by sequential reduction of disulphide bonds with TCEP and alkylation with MMTS [32]. Then, the peptides were extracted and prepared for LC-MS/MS. All LC-MS/MS analyses were performed on an LTQ Orbitrap XL mass spectrometer (Thermo Scientific, San Jose, CA) coupled to an Ultimate 3000 RSLCnano system (Thermo Scientific, formerly Dionex, The Netherlands). Xcalibur raw data files acquired on the LTQ-Orbitrap XL were directly imported into Progenesis LCMS software (Waters Corp., Milford, MA, formerly Non-linear dynamics, Newcastle upon Tyne, UK) for peak detection and alignment. Data were analyzed using the Mascot search engine. Five replicates were analyzed for each sample type (N = 5). Statistical analyses were performed using ANOVA and only fold-changes in proteins with a p-value less than 0.05 were considered significant.

### Data mining

To firmly establish the clinical relevance of our results from the quantitative proteomics analysis of mammospheres, we re-analyzed the transcriptional profiles of epithelial breast cancer cells and adjacent tumor stromal cells that were physically separated by laser-capture microdissection (from N=28 human breast cancer patients) [29, 30].

## References

[1] Easwaran H, Tsai HC, Baylin SB (2014). Cancer epigenetics: tumor heterogeneity, plasticity of stem-like states, and drug resistance. Molecular cell.

[2] Xin H, Kong Y, Jiang X, Wang K, Qin X, Miao ZH, Zhu Y, Tan W (2013). Multi-drug-resistant cells enriched from chronic myeloid leukemia cells by Doxorubicin possess tumor-initiating-cell properties. Journal of pharmacological sciences.

[3] Duggal R, Minev B, Geissinger U, Wang H, Chen NG, Koka PS, Szalay AA (2013). Biotherapeutic approaches to target cancer stem cells. Journal of stem cells.

[4] Sinha N, Mukhopadhyay S, Das DN, Panda PK, Bhutia SK (2013). Relevance of cancer initiating/stem cells in carcinogenesis and therapy resistance in oral cancer. Oral oncology.

[5] Scopelliti A, Cammareri P, Catalano V, Saladino V, Todaro M, Stassi G (2009). Therapeutic implications of Cancer Initiating Cells. Expert opinion on biological therapy.

[6] Chandler JM, Lagasse E (2010). Cancerous stem cells: deviant stem cells with cancer-causing misbehavior. Stem cell research & therapy.

[7] Zhang M, Rosen JM (2006). Stem cells in the etiology and treatment of cancer. Current opinion in genetics & development.

[8] Shaw FL, Harrison H, Spence K, Ablett MP, Simoes BM, Farnie G, Clarke RB (2012). A detailed mammosphere assay protocol for the quantification of breast stem cell activity. Journal of mammary gland biology and neoplasia.

[9] Ponti D, Costa A, Zaffaroni N, Pratesi G, Petrangolini G, Coradini D, Pilotti S, Pierotti MA, Daidone MG (2005). Isolation and *in vitro* propagation of tumorigenic breast cancer cells with stem/progenitor cell properties. Cancer research.

[10] Moore N, Lyle S (2011). Quiescent, slow-cycling stem cell populations in cancer: a review of the evidence and discussion of significance. Journal of oncology.

[11] Chen MS, Woodward WA, Behbod F, Peddibhotla S, Alfaro MP, Buchholz TA, Rosen JM (2007). Wnt/beta-catenin mediates radiation resistance of Sca1+ progenitors in an immortalized mammary gland cell line. Journal of cell science.

[12] Fillmore CM, Kuperwasser C (2008). Human breast cancer cell lines contain stem-like cells that self-renew, give rise to phenotypically diverse progeny and survive chemotherapy. Breast cancer research : BCR.

[13] Dean M (2009). ABC transporters, drug resistance, and cancer stem cells. Journal of mammary gland biology and neoplasia.

[14] Bao S, Wu Q, McLendon RE, Hao Y, Shi Q, Hjelmeland AB, Dewhirst MW, Bigner DD, Rich JN (2006). Glioma stem cells promote radioresistance by preferential activation of the DNA damage response. Nature.

[15] Phillips TM, McBride WH, Pajonk F (2006). The response of CD24(−/low)/CD44+ breast cancer-initiating cells to radiation. Journal of the National Cancer Institute.

[16] Jackson TR, Salmina K, Huna A, Inashkina I, Jankevics E, Riekstina U, Kalnina Z, Ivanov A, Townsend PA, Cragg MS, Erenpreisa J (2013). DNA damage causes TP53-dependent coupling of self-renewal and senescence pathways in embryonal carcinoma cells. Cell cycle.

[17] Salmina K, Jankevics E, Huna A, Perminov D, Radovica I, Klymenko T, Ivanov A, Jascenko E, Scherthan H, Cragg M, Erenpreisa J (2010). Up-regulation of the embryonic self-renewal network through reversible polyploidy in irradiated p53-mutant tumour cells. Experimental cell research.

[18] Lagadec C, Vlashi E, Della Donna L, Dekmezian C, Pajonk F (2012). Radiation-induced reprogramming of breast cancer cells. Stem cells.

[19] Abubaker K, Latifi A, Luwor R, Nazaretian S, Zhu H, Quinn MA, Thompson EW, Findlay JK, Ahmed N (2013). Short-term single treatment of chemotherapy results in the enrichment of ovarian cancer stem cell-like cells leading to an increased tumor burden. Molecular cancer.

[20] Nathans D (1964). Puromycin Inhibition of Protein Synthesis: Incorporation of Puromycin into Peptide Chains. Proceedings of the National Academy of Sciences of the United States of America.

[21] Popovich IG, Anisimov VN, Zabezhinski MA, Semenchenko AV, Tyndyk ML, Yurova MN, Blagosklonny MV (2014). Lifespan extension and cancer prevention in HER-2/neu transgenic mice treated with low intermittent doses of rapamycin. Cancer biology & therapy.

[22] Mercier I, Camacho J, Titchen K, Gonzales DM, Quann K, Bryant KG, Molchansky A, Milliman JN, Whitaker-Menezes D, Sotgia F, Jasmin JF, Schwarting R, Pestell RG, Blagosklonny MV, Lisanti MP (2012). Caveolin-1 and accelerated host aging in the breast tumor microenvironment: chemoprevention with rapamycin, an mTOR inhibitor and anti-aging drug. Am J Pathol.

[23] Gao B, Roux PP (2014). Translational control by oncogenic signaling pathways. Biochimica et biophysica acta.

[24] Liu H, Zhang W, Wang K, Wang X, Yin F, Li C, Wang C, Zhao B, Zhong C, Zhang J, Peng F, Bi Y, Shen C, Hou X, Zhang D, Liu Y (2014). Methionine and cystine double deprivation stress suppresses glioma proliferation via inducing ROS/autophagy. Toxicology letters.

[25] Sinha R, Cooper TK, Rogers CJ, Sinha I, Turbitt WJ, Calcagnotto A, Perrone CE, Richie JP (2014). Dietary methionine restriction inhibits prostatic intraepithelial neoplasia in TRAMP mice. The Prostate.

[26] Cavuoto P, Fenech MF (2012). A review of methionine dependency and the role of methionine restriction in cancer growth control and life-span extension. Cancer treatment reviews.

[27] Saleh AD, Simone BA, Palazzo J, Savage JE, Sano Y, Dan T, Jin L, Champ CE, Zhao S, Lim M, Sotgia F, Camphausen K, Pestell RG, Mitchell JB, Lisanti MP, Simone NL (2013). Caloric restriction augments radiation efficacy in breast cancer. Cell cycle.

[28] Champ CE, Baserga R, Mishra MV, Jin L, Sotgia F, Lisanti MP, Pestell RG, Dicker AP, Simone NL (2013). Nutrient restriction and radiation therapy for cancer treatment: when less is more. The oncologist.

[29] Casey T, Bond J, Tighe S, Hunter T, Lintault L, Patel O, Eneman J, Crocker A, White J, Tessitore J, Stanley M, Harlow S, Weaver D, Muss H, Plaut K (2009). Molecular signatures suggest a major role for stromal cells in development of invasive breast cancer. Breast cancer research and treatment.

[30] Sotgia F, Whitaker-Menezes D, Martinez-Outschoorn UE, Salem AF, Tsirigos A, Lamb R, Sneddon S, Hulit J, Howell A, Lisanti MP (2012). Mitochondria “fuel” breast cancer metabolism: fifteen markers of mitochondrial biogenesis label epithelial cancer cells, but are excluded from adjacent stromal cells. Cell cycle.

[31] Francipane MG, Lagasse E (2013). Selective targeting of human colon cancer stem-like cells by the mTOR inhibitor Torin-1. Oncotarget.

[32] Lamb R, Harrison H, Hulit J, Smith DL, Lisanti MP, Sotgia F (2014). Mitochondria as new therapeutic targets for eradicating cancer stem cells: Quantitative proteomics and functional validation via MCT1/2 inhibition. Oncotarget.

[33] Dang CV, O'Donnell KA, Zeller KI, Nguyen T, Osthus RC, Li F (2006). The c-Myc target gene network. Seminars in cancer biology.

[34] Gomez-Roman N, Felton-Edkins ZA, Kenneth NS, Goodfellow SJ, Athineos D, Zhang J, Ramsbottom BA, Innes F, Kantidakis T, Kerr ER, Brodie J, Grandori C, White RJ (2006). Activation by c-Myc of transcription by RNA polymerases I, II and III. Biochemical Society symposium.

[35] Iritani BM, Eisenman RN (1999). c-Myc enhances protein synthesis and cell size during B lymphocyte development. Proceedings of the National Academy of Sciences of the United States of America.

[36] Coller HA, Grandori C, Tamayo P, Colbert T, Lander ES, Eisenman RN, Golub TR (2000). Expression analysis with oligonucleotide microarrays reveals that MYC regulates genes involved in growth, cell cycle, signaling, and adhesion. Proceedings of the National Academy of Sciences of the United States of America.

[37] Greasley PJ, Bonnard C, Amati B (2000). Myc induces the nucleolin and BN51 genes: possible implications in ribosome biogenesis. Nucleic acids research.

[38] Guo QM, Malek RL, Kim S, Chiao C, He M, Ruffy M, Sanka K, Lee NH, Dang CV, Liu ET (2000). Identification of c-myc responsive genes using rat cDNA microarray. Cancer research.

[39] Ruggero D (2009). The role of Myc-induced protein synthesis in cancer. Cancer research.

[40] Barna M, Pusic A, Zollo O, Costa M, Kondrashov N, Rego E, Rao PH, Ruggero D (2008). Suppression of Myc oncogenic activity by ribosomal protein haploinsufficiency. Nature.

[41] Proud CG (2007). Amino acids and mTOR signalling in anabolic function. Biochemical Society transactions.

[42] Proud CG (2007). Cell signaling. mTOR, unleashed. Science.

[43] Iadevaia V, Wang X, Yao Z, Foster LJ, Proud CG (2012). Evaluation of mTOR-regulated mRNA translation. Methods in molecular biology.

[44] Wang X, Proud CG (2006). The mTOR pathway in the control of protein synthesis. Physiology.

[45] Iadevaia V, Zhang Z, Jan E, Proud CG (2012). mTOR signaling regulates the processing of pre-rRNA in human cells. Nucleic acids research.

[46] Proud CG (2011). mTOR Signalling in Health and Disease. Biochemical Society transactions.

[47] Averous J, Proud CG (2006). When translation meets transformation: the mTOR story. Oncogene.

[48] Vogt PK (2001). PI 3-kinase, mTOR, protein synthesis and cancer. Trends in molecular medicine.

[49] Blume-Jensen P, Hunter T (2001). Oncogenic kinase signalling. Nature.

[50] Burnett PE, Barrow RK, Cohen NA, Snyder SH, Sabatini DM (1998). RAFT1 phosphorylation of the translational regulators p70 S6 kinase and 4E-BP1. Proceedings of the National Academy of Sciences of the United States of America.

[51] Brunn GJ, Hudson CC, Sekulic A, Williams JM, Hosoi H, Houghton PJ, Lawrence JC, Abraham RT (1997). Phosphorylation of the translational repressor PHAS-I by the mammalian target of rapamycin. Science.

[52] Jefferies HB, Fumagalli S, Dennis PB, Reinhard C, Pearson RB, Thomas G (1997). Rapamycin suppresses 5′TOP mRNA translation through inhibition of p70s6k. The EMBO journal.

[53] Shiraki N, Shiraki Y, Tsuyama T, Obata F, Miura M, Nagae G, Aburatani H, Kume K, Endo F, Kume S (2014). Methionine metabolism regulates maintenance and differentiation of human pluripotent stem cells. Cell metabolism.

[54] Matsuura K, Kodama F, Sugiyama K, Shimizu T, Hagiwara N, Okano T (2014). Elimination of Remaining Undifferentiated Induced Pluripotent Stem Cells in the Process of Human Cardiac Cell Sheet Fabrication Using a Methionine-Free Culture Condition. Tissue engineering Part C, Methods.

[55] Sugimura T, Birnbaum SM, Winitz M, Greenstein JP (1959). Quantitative nutritional studies with water-soluble, chemically defined diets. VIII. The forced feeding of diets each lacking in one essential amino acid. Archives of biochemistry and biophysics.

[56] Breillout F, Hadida F, Echinard-Garin P, Lascaux V, Poupon MF (1987). Decreased rat rhabdomyosarcoma pulmonary metastases in response to a low methionine diet. Anticancer research.

[57] Guo H, Lishko VK, Herrera H, Groce A, Kubota T, Hoffman RM (1993). Therapeutic tumor-specific cell cycle block induced by methionine starvation *in vivo*. Cancer research.

[58] Theuer RC (1971). Effect of essential amino acid restriction on the growth of female C57BL mice and their implanted BW10232 adenocarcinomas. The Journal of nutrition.

[59] Halpern BC, Clark BR, Hardy DN, Halpern RM, Smith RA (1974). The effect of replacement of methionine by homocystine on survival of malignant and normal adult mammalian cells in culture. Proceedings of the National Academy of Sciences of the United States of America.

[60] Mecham JO, Rowitch D, Wallace CD, Stern PH, Hoffman RM (1983). The metabolic defect of methionine dependence occurs frequently in human tumor cell lines. Biochemical and biophysical research communications.

[61] Cao WX, Ou JM, Fei XF, Zhu ZG, Yin HR, Yan M, Lin YZ (2002). Methionine-dependence and combination chemotherapy on human gastric cancer cells *in vitro*. World journal of gastroenterology : WJG.

[62] Hoshiya Y, Guo H, Kubota T, Inada T, Asanuma F, Yamada Y, Koh J, Kitajima M, Hoffman RM (1995). Human tumors are methionine dependent *in vivo*. Anticancer research.

[63] Poirson-Bichat F, Goncalves RA, Miccoli L, Dutrillaux B, Poupon MF (2000). Methionine depletion enhances the antitumoral efficacy of cytotoxic agents in drug-resistant human tumor xenografts. Clinical cancer research : an official journal of the American Association for Cancer Research.

[64] Barja G (2014). The mitochondrial free radical theory of aging. Progress in molecular biology and translational science.

[65] Sanchez-Roman I, Barja G (2013). Regulation of longevity and oxidative stress by nutritional interventions: role of methionine restriction. Experimental gerontology.

[66] Johnson JE, Johnson FB (2014). Methionine restriction activates the retrograde response and confers both stress tolerance and lifespan extension to yeast, mouse and human cells. PloS one.

[67] Koziel R, Ruckenstuhl C, Albertini E, Neuhaus M, Netzberger C, Bust M, Madeo F, Wiesner RJ, Jansen-Durr P (2014). Methionine restriction slows down senescence in human diploid fibroblasts. Aging cell.

[68] Brown-Borg HM, Rakoczy SG, Wonderlich JA, Rojanathammanee L, Kopchick JJ, Armstrong V, Raasakka D (2014). Growth hormone signaling is necessary for lifespan extension by dietary methionine. Aging cell.

[69] Huang T, Yang B, Zheng J, Li G, Wahlqvist ML, Li D (2012). Cardiovascular disease mortality and cancer incidence in vegetarians: a meta-analysis and systematic review. Annals of nutrition & metabolism.

[70] Orlich MJ, Singh PN, Sabate J, Jaceldo-Siegl K, Fan J, Knutsen S, Beeson WL, Fraser GE (2013). Vegetarian dietary patterns and mortality in Adventist Health Study 2. JAMA internal medicine.

[71] Rohrmann S, Overvad K, Bueno-de-Mesquita HB, Jakobsen MU, Egeberg R, Tjonneland A, Nailler L, Boutron-Ruault MC, Clavel-Chapelon F, Krogh V, Palli D, Panico S, Tumino R, Ricceri F, Bergmann MM, Boeing H (2013). Meat consumption and mortality--results from the European Prospective Investigation into Cancer and Nutrition. BMC medicine.

[72] McCarty MF, Barroso-Aranda J, Contreras F (2009). The low-methionine content of vegan diets may make methionine restriction feasible as a life extension strategy. Medical hypotheses.

[73] Ornish D, Weidner G, Fair WR, Marlin R, Pettengill EB, Raisin CJ, Dunn-Emke S, Crutchfield L, Jacobs FN, Barnard RJ, Aronson WJ, McCormac P, McKnight DJ, Fein JD, Dnistrian AM, Weinstein J (2005). Intensive lifestyle changes may affect the progression of prostate cancer. The Journal of urology.

[74] Epner DE, Morrow S, Wilcox M, Houghton JL (2002). Nutrient intake and nutritional indexes in adults with metastatic cancer on a phase I clinical trial of dietary methionine restriction. Nutrition and cancer.

